# miR-155 Regulated Inflammation Response by the SOCS1-STAT3-PDCD4 Axis in Atherogenesis

**DOI:** 10.1155/2016/8060182

**Published:** 2016-10-24

**Authors:** Jinshan Ye, Ruiwei Guo, Yankun Shi, Feng Qi, Chuanming Guo, Lixia Yang

**Affiliations:** ^1^Department of Postgraduate, Third Military Medical University, Chongqing 400038, China; ^2^Department of Cardiology, Kunming General Hospital of Chengdu Military Area, Yunnan 650032, China

## Abstract

Inflammation response plays a critical role in all phases of atherosclerosis (AS). Increased evidence has demonstrated that miR-155 mediates inflammatory mediators in macrophages to promote plaque formation and rupture. However, the precise mechanism of miR-155 remains unclear in AS. Here, we also found that miR-155 and PDCD4 were elevated in the aortic tissue of atherosclerotic mice and ox-LDL treated RAW264.7 cells. Further studies showed that miR-155 not only directly inhibited SOCS1 expression, but also increased the expression of p-STAT and PDCD4, as well as the production of proinflammation mediators IL-6 and TNF-*α*. Downregulation of miR-155 and PDCD4 and upregulation of SOCS1 obviously decreased the IL-6 and TNF-*α* expression. In addition, inhibition of miR-155 levels in atherosclerotic mice could notably reduce the IL-6 and TNF-*α* level in plasma and aortic tissue, accompanied with increased p-STAT3 and PDCD4 and decreased SOCS1. Thus, miR-155 might mediate the inflammation in AS via the SOCS1-STAT3-PDCD4 axis. These results provide a rationale for intervention of intracellular miR-155 as possible antiatherosclerotic targets.

## 1. Introduction

Atherosclerosis (AS) remains a major cause of mortality worldwide, causing acute cardiovascular events and chronic damage, including ischemic heart disease and ischemic stroke [1]. It is well known that inflammation plays an important role in all phases of AS [2, 3]. In the initiation and progression of AS, many immune cells, especially M1-type macrophages, are recruited to the arterial wall where they produce and secrete extensive amounts of inflammation mediators and chemokines and promote the formation of plaque [3] and plaque rupture [4]. This leads to plaque instability, thrombosis, and, finally, cardiovascular events [5].

MicroRNAs (miRNAs), which are a class of 18–22 nt small noncoding RNAs, have served as negative regulators of gene expression at a posttranscriptional level [6] and play an important role in cell development, metabolism, proliferation, and apoptosis [7, 8]. miRNAs are involved in the pathogenesis of many diseases, from cancer to cardiovascular disease [9]. Studies have recently found that several miRNAs, such as miR-155 [10] and miR-33 [11], are involved in the initiation and progress of AS. In particular, miR-155, located within a region known as the B-cell integration cluster (BIC) in the genome, plays a key role in innate immunity [12, 13]. A broad range of inflammatory factors, including ox-LDL, stimulate macrophages, and they in turn regulate the expression of inflammatory factors to enhance cellular inflammatory response [13]. In addition, other results showed that miR-155 is mainly expressed in macrophages and SMCs in late atherosclerotic lesions, and lesional macrophages are an especially abundant source of miR-155 [14]. How the precision mechanism of miR-155 regulated the formation of macrophage-derived foam cells during early atherogenesis is still not clear.

Historically, programmed cell death 4 (PDCD4) was always notably reduced or deficient in various tumors [15–17]. PDCD4 activation was involved in the apoptosis of cancer cells [17, 18] and was therefore considered a tumor suppressor [19]. Interestingly, Billiard et al. showed that PDCD4 deficient mice were resistant to inflammatory diseases [20]. Zhong et al. found that PDCD4 was involved in allergic pulmonary inflammation through regulated macrophage alternative activation [21]. Other studies showed that PDCD4 improved the inflammatory response via nuclear factor-*κ*B (NF-*κ*B) activation and inhibition in the production of interleukin- (IL-) 10 [22, 23]. Liang et al. demonstrated that PDCD4 was elevated in atherosclerosis mice and in the foam cells, and inhibition of PDCD4 could suppress the inflammation mediator, such as IL-6 and TNF-*α* [24]. Other studies showed that PDCD4 deficiency in mice increased the expression of IL-10 in macrophages and led to a decrease in atherosclerotic lesions in ApoE^−/−^ mice who were fed high fat diets (HFD) [25]. These results suggest the potential role of PDCD4 as a novel therapeutic target in the clinical treatment of atherosclerosis. Although several reports showed that PDCD4 was directly regulated by miR-21 and miR-16 in macrophages, whether miR-155 regulated the PDCD4 is unclear.

In this study, we explored elevated miR-155 and PDCD4 in the aortic tissue of atherosclerotic mice and ox-LDL treated RAW264.7 cells. We also showed that miR-155 directly inhibits SOCS1 expression and increases the expression of p-STAT and PDCD4, thereby promoting the production of proinflammation mediators. Knocking down of miR-155, SOCS1 overexpression, and downregulation of PDCD4 notably affected the production and release of the inflammation mediator. Furthermore, inhibition of the miR-155 level in atherosclerotic mice also elevated the SOCS1 expression and decreased the expression of p-STAT3, PDCD4, and proinflammation cytokine levels. In summary, we determined that miR-155 might promote an inflammation response of AS through the SOCS1-STAT3-PDCD4 axis.

## 2. Methods

### 2.1. Cell Culture and Treatment

A macrophage Raw264.7 cell line was purchased from American Type Culture Collection and maintained in DMEM medium with 10% FBS and 1% antibiotics. These cells were treated with 5 *μ*g/mL, 10 *μ*g/mL, and 20 *μ*g/mL ox-LDL for 24 h or 20 *μ*g/mL ox-LDL from 6–12 h. STAT3, PDCD4 siRNA, anti-miR-155, and anti-NC were transfected into macrophages for 24 h, respectively, and these cells were exposed with 20 *μ*g/mL ox-LDL for 24 h. Thereafter, the medium was changed to normal culture medium to continue further study. These siRNA target sites were showed in Supplemental Tables S1 and S2 (in Supplementary Material available online at http://dx.doi.org/10.1155/2016/8060182).

### 2.2. Total RNA Isolation and Real-Time PCR

Total RNA from cells and aortic tissue was extracted by using the TRIzol reagent according to the manufacturer's protocol. Reverse transcription polymerase chain reaction (RT-PCR) was conducted in a two-step process using an RNA PCR Kit according to the manufacturer's instructions. The primers of miR-155, SOCS1, STAT3, and PDCD4 showed in Supplemental Table S3. Each PCR amplification was performed under the following conditions: 95°C for 10 min, 95°C for 20 s, and 60°C for 20 s at the annealing temperature through 35 cycles.

### 2.3. Cytokine Assay by ELISA

The plasma of IL-10, IL-6, and TNF-*α* level was detected by ELISA according to manufacturer's instructions. The details had been shown in the Lei and colleagues study [26].

### 2.4. Western Blot

Macrophage cells and aortic tissue were collected and homogenized with lysis buffer (Pierce), and then the concentrations of total protein were determined using a BCA kit (Pierce). Immunoblotting was conducted following procedures outlined in a study [27]. Rabbit polyclonal anti-mouse phospho-STAT3 (p-STAT3), mouse monoclonal anti-PDCD4, and rabbit polyclonal SOCS1 were obtained from Abcam, Inc.

### 2.5. Constructed SOCS1 Adenoviruses

SOCS1 wild-type (WT) adenoviruses were constructed as outlined in the study by Galam et al. [28]. Briefly, 293T cells were transfected with SOCS1, cDNA vectors, and a packing system (SystemViraPower™ Adenoviruses Expression Systems, Invitrogen). After 293T cells were transfected, all plasmids and adenovirus-containing media were harvested at 48 and 72 h. Thereafter, the virus was purified by CsCl gradients.

### 2.6. Luciferase Reporter Assay

The 3′-UTR of SOCS1 was synthesized and annealed and then inserted into the SpeI and HindIII sites of pMIR-reporter luciferase vector downstream of the stop codon of the gene for luciferase. For its mutagenesis, the sequences complementary to the binding site of miR-155 in the 3′-UTR (SOCS1: AGCAUUA) were replaced by AGCUAAU. These constructs were validated by sequencing. 293T cells were seeded into a 24-well plate for a luciferase assay. After cultured overnight, cells were cotransfected with the wild-type or mutated plasmid, pRL-TK plasmid, and equal amounts of miR-155 or miR-NC. The pRL-TK control vector was also transfected as a control. Luciferase assays were performed 24 h after transfection using the Dual Luciferase® Reporter Assay System. Firefly and Renilla reniformis luciferase activities were measured 24 h later. Experiments were performed in three independent replicates.

### 2.7. Animal Experiments

Male C57 wild-type (WT) mice and ApoE^−/−^ mice were purchased from the Model Animal Research Center of Nanjing University. ApoE^−/−^ mice (6 weeks, male) were fed HFDs (21% fat, 1.25% cholesterol) for 1 week and then randomized into two groups (*n* = 10 mice, resp.): control antagomiR-injected and antagomiR-155-injected groups. The mice received two subcutaneous injections of 25 mg/kg antagomiR-155 or antagomiR for the first week, spaced 36 days apart, and then weekly injections of 25 mg/kg antagomiR or antagomiR-155 thereafter for 9 weeks, at which point injections of antagomiR or antagomiR-155 were stopped. They were both still fed, however, with an HFD for 1 week. Aortic roots of mice were embedded in OCT medium and frozen immediately. All animal experiments were carried out in accordance with the National Institutes of Health* Guide for the Care and Use of Laboratory Animals* and were approved by the Biological Research Ethics Committee of the Institute of Health Sciences.

### 2.8. Atherosclerosis Analysis

Thoracoabdominal aortas were fixed by 10% formaldehyde (sigma) for 12 h, and then the plaques were stained with Oil Red O staining. The collagen size of atherosclerotic plaques was analyzed by using Sirius red and fast green collagen staining.

### 2.9. Statistical Analysis

Data were expressed as mean ± standard error (SE) and were achieved via at least three independent experiments. Two-tailed Student's* t*-test and one-way analyses of variance (ANOVA) were performed. The significant statistical difference was defined according to *p* < 0.05.

## 3. Results

### 3.1. miR-155 Induced by ox-LDL in Macrophages RAW264.7 Cells and Increased in the Aortic Tissue of ApoE^−/−^ Mice

**Figure 1:**
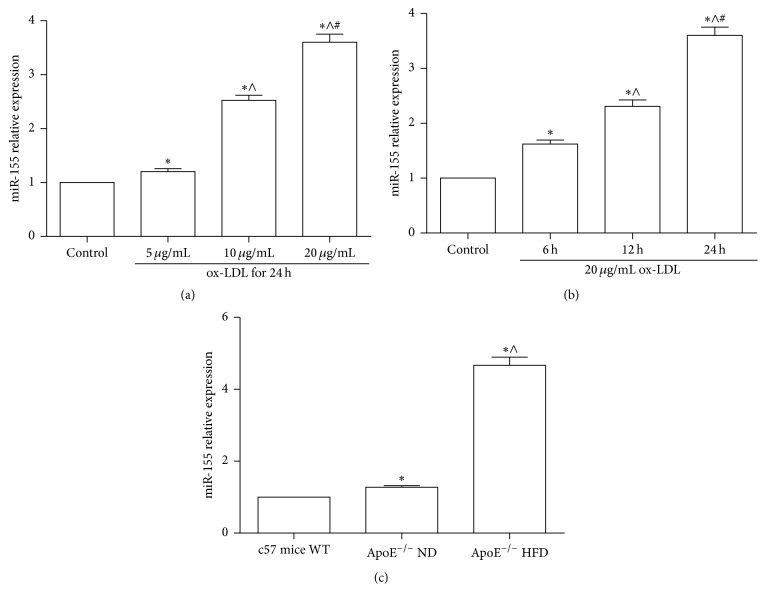
The relative expression of miR-155 in macrophages RAW264.7 cells and the aortic tissue of ApoE^−/−^ mice. (a) qPCR analyzed the expression of miR-155 in RAW264.7 cells when macrophages were treated by ox-LDL for 24 h at the indicated dose: ^*∗*^ *p* < 0.05, relative to control, ^∧^ *p* < 0.05, relative to 5 *μ*g/mL ox-LDL group, ^#^ *p* < 0.05, relative to 10 *μ*g/mL ox-LDL group. (b) qPCR detected the level of miR-155 in macrophages, which were treated with 20 *μ*g/mL ox-LDL at the indicated time: ^*∗*^ *p* < 0.05, relative to control, ^∧^ *p* < 0.05, relative to 6 h group, ^#^ *p* < 0.05, relative to 12 h group. (c) Expression of miR-155 in aortic tissue of ND-fed wild-type (WT) c57 mice, ND-fed ApoE^−/−^ mice (ApoE^−/−^ ND), and HFD-fed ApoE^−/−^ mice (ApoE^−/−^ HFD) was determined by qPCR (*n* = 4): ^*∗*^ *p* < 0.05, relative to c57 mice WT; ^∧^ *p* < 0.05, relative to ApoE^−/−^ ND.

In this study, we investigated the expression of miR-155 after RAW264.7 cells were treated by ox-LDL at the indicated dose and time (Figures 1(a) and 1(b)). The data showed that ox-LDL gradually increased the expression of miR-155 with increased concentrations from 5 *μ*g/mL to 20 *μ*g/mL treatment with macrophages for 24 h, compared to control (*p* < 0.05), and the expression of miR-155 was peaked while cells were treated with 20 *μ*g/mL ox-LDL. The result also showed that miR-155 was significantly higher after the RAW264.7 cells (treated with 20 *μ*g/mL ox-LDL for 6 h, 12 h, and 24 h) than in control (*p* < 0.05). Therefore, ox-LDL induced miR-155 in a dose- and time-dependent manner. Moreover, we evaluated the miR-155 expression in the aortic tissue of ApoE^−/−^ mice with normal food (ND) and HFD. The results revealed that the expression of miR-155 elevated in aortic tissue of ApoE^−/−^ ND and ApoE^−/−^ HFD, compared to the c57 mice (*p* < 0.05), and it was notably higher in ApoE^−/−^ HFD than in ApoE^−/−^ ND (*p* < 0.05). These data suggest that miR-155 was induced by ox-LDL, and it could be involved in the pathogenesis of atherosclerosis [29].

### 3.2. ox-LDL Mediated the Production of Cytokines in RAW264.7 Cells and Plasma Levels of These Cytokines Changed in Atherosclerotic Mice

**Figure 2:**
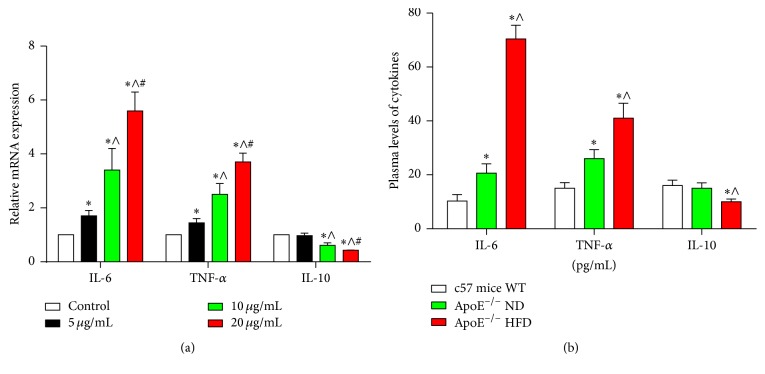
The levels of cytokines IL-6, TNF-*α*, and IL-10 in RAW264.7 cells after ox-LDL treatment and atherosclerotic mice. (a) ox-LDL induced the expression of IL-6, TNF-*α*, and IL-10 for 24 h at indicated dose was determined by qPCR: ^*∗*^ *p* < 0.05, relative to control, ^∧^ *p* < 0.05, relative to 5 *μ*g/mL ox-LDL group; ^#^ *p* < 0.05, relative to 10 *μ*g/mL ox-LDL group. (b) The plasma levels of IL-6, TNF-*α*, and IL-10 of c57 mice WT, ApoE^−/−^ ND, and ApoE^−/−^ HFD were detected by the ELISA kit (*n* = 6): ^*∗*^ *p* < 0.05, relative to c57 mice WT; ^∧^ *p* < 0.05, relative to ApoE^−/−^ ND.

The production and release of inflammation mediators by macrophages played a critical role in AS [27, 30, 31]. In our study, we showed that the expression of IL-6 and TNF-*α* mRNA was increased and IL-10 mRNA expression was inhibited by increasing ox-LDL concentration, compared to the control group in RAW264.7 cells (Figure 2(a)). In addition, the level of plasma IL-6 and TNF-*α* was higher in the ApoE^−/−^ ND and ApoE^−/−^ HFD groups than in the c57 mice (*p* < 0.05). These were notably elevated in ApoE^−/−^ HFD compared with ApoE^−/−^ ND (*p* < 0.05) (Figure 2(b)). On the contrary, the plasma IL-10 level was significantly reduced in ApoE^−/−^ HFD, compared to the ApoE^−/−^ ND and c57 mice group (*p* < 0.05); however, there was no difference between the ApoE^−/−^ ND and c57 mice group (*p* > 0.05) (Figure 2(b)).

### 3.3. PDCD4 Regulated by miR-155 In Vitro

**Figure 3:**
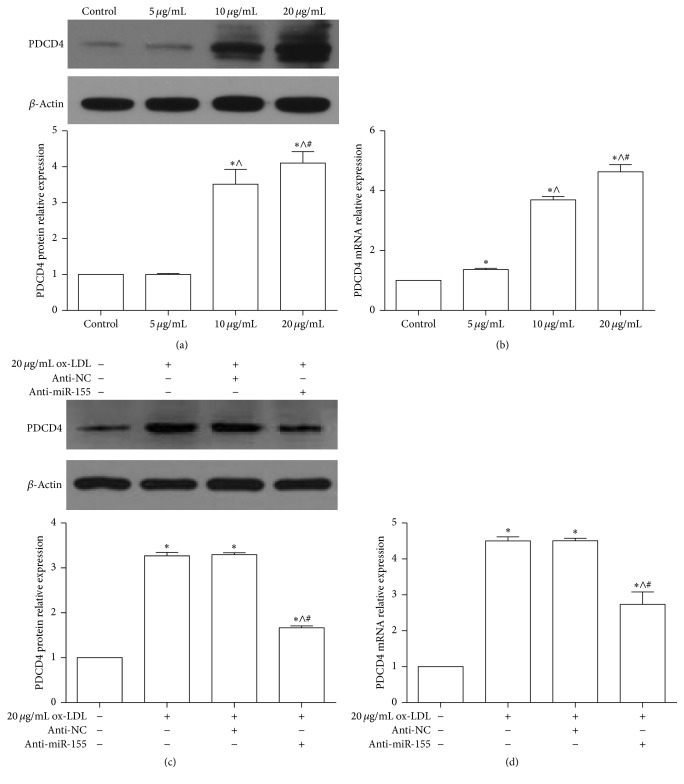
The relative expression of PDCD4 in macrophages RAW264.7 cells. (a and b) Western blot and qPCR analyzed the PDCD4 protein and mRNA expression after indicated ox-LDL treated macrophages RAW264.7 cells for 24 h. Representative bands show the protein expression of PDCD4 (the upper panel), *β*-actin (the middle panel), and the histograms showing quantification of PDCD4 bands normalization relative to *β*-actin expression in (a): ^*∗*^ *p* < 0.05 versus control, ^∧^ *p* < 0.05 versus 5 *μ*g/mL group, and ^#^ *p* < 0.05 versus 10 *μ*g/mL group. (c and d) Western blot and qPCR analyzed the PDCD4 protein and mRNA expression while RAW264.7 cells were treated by 20 *μ*g/mL ox-LDL plus anti-miR-155 or anti-NC. 100 pM anti-miR-155 or anti-NC had been transfected into RAW264.7 cells for 24 h, and then they were treated by 20 *μ*g/mL ox-LDL for 24 h. Representative bands show the protein expression of PDCD4 (the upper panel), *β*-actin (the middle panel), and the histograms showing quantification of PDCD4 bands normalized relative to *β*-actin expression in (c): ^*∗*^ *p* < 0.05 versus control, ^∧^ *p* < 0.05 versus 20 *μ*g/mL ox-LDL group, and ^#^ *p* < 0.05 versus 20 *μ*g/mL ox-LDL plus anti-NC group.

Previous studies reveal that knocked down PDCD4 played an important role in attenuating foam cell formation and atherosclerosis in ApoE^−/−^ mice [32]. Consistent with miR-155, PDCD4 expression was increased upon ox-LDL stimulation in a dose-dependent manner (Figures 3(a) and 3(b)). To further investigate the relationship between miR-155 and PDCD4, RAW264.7 cells were transfected with anti-miR-155 and miR-NC and then challenged with ox-LDL. These results showed that the inhibition of miR-155 could reduce the expression of PDCD4 while RAW264.7 cells are treated by ox-LDL (Figures 3(c) and 3(d)). Together, these results suggest that miR-155 could regulate PDCD4 expression in macrophages; however, the mechanism was unclear.

### 3.4. Downregulation of PDCD4 and miR-155 Mediated Production of Cytokines IL-6, TNF-*α*, and IL-10 in RAW264.7 Cells

**Figure 4:**
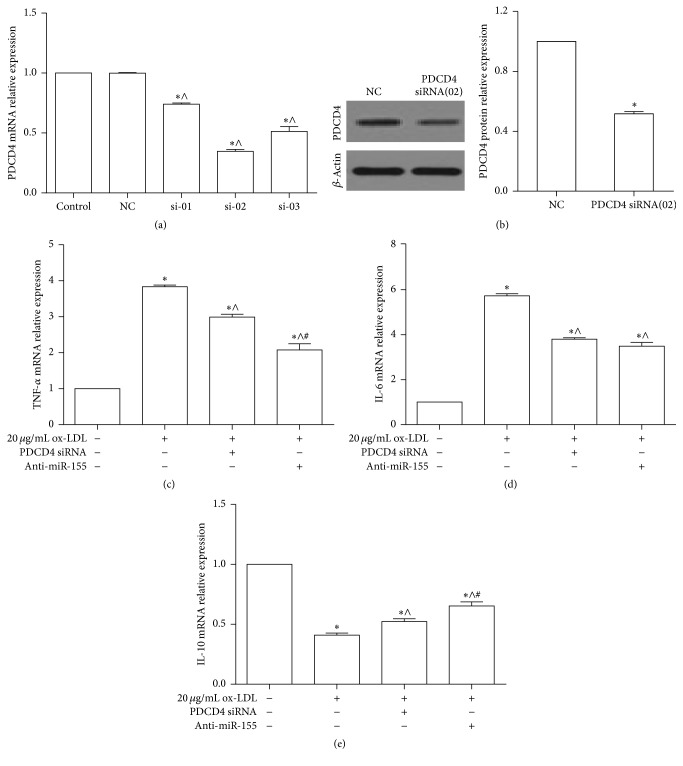
Downregulation of PDCD4 and miR-155 regulated the expression of IL-6, TNF-*α*, and IL-10. (a) Knocking down the expression of PDCD4 mRNA by PDCD4 siRNA in macrophages RAW264.7 cells. The relative expression of PDCD4 mRNA was detected by qPCR after 50pM PDCD4 siRNA, including si-01, si-02, and si-03, and negative control (NC) was transfected into RAW264.7 cells for 48 h, respectively: ^*∗*^ *p* < 0.05 versus control, ^∧^ *p* < 0.05 versus NC group. (b) Western blot analyzed the protein level of PDCD4 when RAW264.7 cells had been treated by 50pM PDCD4 siRNA (si-02) and NC for 48 h. Representative bands show the protein expression of PDCD4 (the upper bands), *β*-actin (the lower bands) in left panel, and the histograms in right panel showing quantification of PDCD4 bands normalization relative to *β*-actin expression in (b): ^*∗*^ *p* < 0.05 versus NC group. (c, d, and e) qPCR analyzed the mRNA expression of TNF-*α*, IL-6, and IL-10 in RAW264.7 cells. They were transfected into the anti-miR-155 or PDCD4 siRNA for 24 h and then treated by ox-LDL for 24 h: ^*∗*^ *p* < 0.05 versus control, ^∧^ *p* < 0.05 versus 20 *μ*g/mL ox-LDL group, and ^#^ *p* < 0.05 versus 20 *μ*g/mL ox-LDL plus PDCD4 siRNA group. The data are presented as the mean ± SE of three separate experiments.

PDCD4 and miR-155 played important roles in the regulation of inflammation response [24, 33–35]. Three PDCD4 siRNA oligos were transfected into RAW264.7 cells to evaluate the suppression effective of PDCD4 expression. This data showed that PDCD4 siRNA oligo (02) was more effective in knocking down PDCD4 than other siRNA oligos (Figure 4(a)). Moreover, the protein expression of PDCD4 was significantly decreased after cells were treated with PDCD4 siRNA oligo (02) (Figure 4(b)). In our study, the inflammation mediators were detected after PDCD4 and miR-155 were knocked down in RAW264.7 cells. qPCR detected demonstrated that downregulation of PDCD4 and miR-155 could partly reverse the elevation of IL-6 and TNF-*α* mRNA expression, which was induced by 20 *μ*g/mL ox-LDL (Figures 4(c) and 4(d)). On the contrary, knocked down PDCD4 and miR-155 obviously increased the expression of IL-10 mRNA when cells were exposed to ox-LDL. These data suggested that miR-155 mediated the production of the inflammation mediator via PDCD4.

### 3.5. miR-155 Regulated PDCD4 via SOCS1-STAT3 Signal Pathway

**Figure 5:**
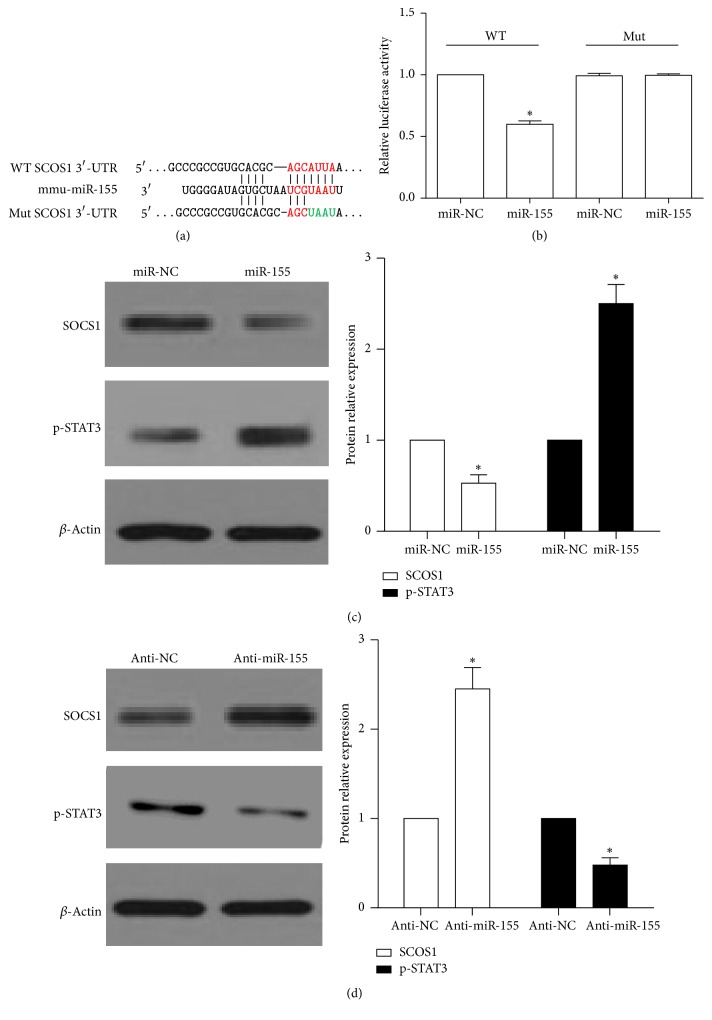
miR-155 regulated directly SOCS1-STAT3 signal pathway. (a) Schematic of the interaction sites of 3′ UTR SOCS1 wild-type (WT) or mutation (Mut) with miR-155. (b) Luciferase reporter assay was used to validate miR-155 binding SOCS1 3′UTR in HEK293FT cells. The luciferase reporter plasmids carrying the WT or Mut 3′UTRof SOCS1 and miR-155 mimic/miR-negative control (NC) were cotransfected into HEK 293FT cells for 24 h, and then luciferase activity was detected: ^*∗*^ *p* < 0.05, relative to miR-NC group. (c) and (d) Western blot analyzed the protein expression of SOCS1 and p-STAT3 after miR-155 mimic, anti-miR-155, and miR-NC was transfected into RAW264.7 cells for 48 h, respectively: ^*∗*^ *p* < 0.05, relative to miR-NC group. The data are presented as the mean ± SE of three separate experiments.

Several studies showed that miR-155 regulated the production of inflammation mediator via direct target [36–38]. In our study, HEK293 cells were cotransfected with the wild-type (WT) or mutated (Mut) SOCS1 luciferase reporter vector, together with miR-155 mimic or miR-NC, for 24 h. Luciferase activity was significantly inhibited in cells transfected with WT SOCS1 and miR-155 mimic, but not in cells transfected with mutation SOCS1 and miR-155 mimic (Figures 5(a) and 5(b)). This data demonstrated that SOCS1 was a direct target of miR155 [38]. To further confirm the effect of miR-155 regulation SOCS1 in RAW264.7 cells, we detected the SOCS1 and its downstream p-STAT3 protein expression after cells were transfected with miR-155 mimic or anti-miR-155. Western blotting analysis indicated that the expression of SOCS1 protein was downregulated and p-STAT3 was upregulated in miR-155-treated RAW264.7 cells (Figure 5(c)). On the contrary, the SOCS1 expression was increased and p-STAT3 was decreased in anti-miR-155 treated cells (Figure 5(d)).

**Figure 6:**
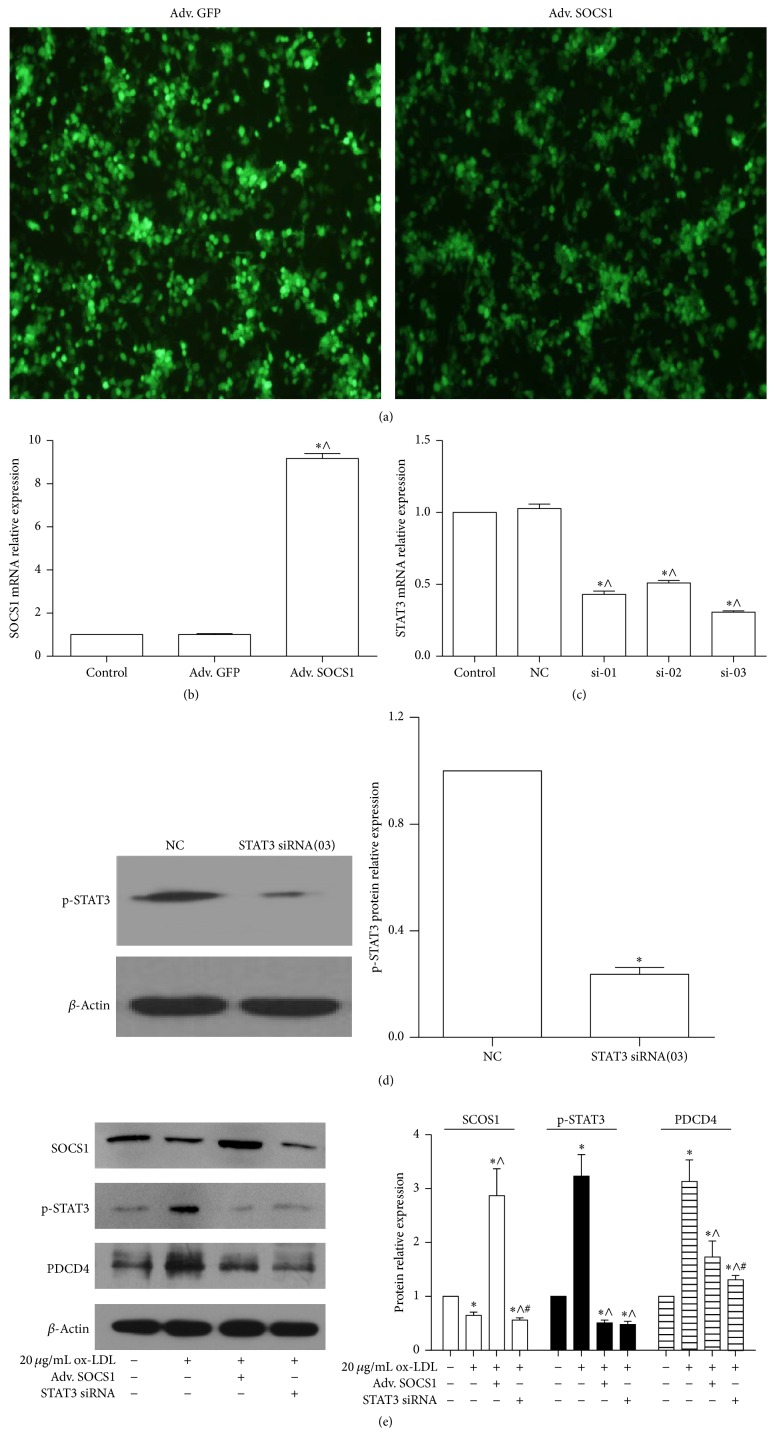
SOCS1-STAT3 pathway regulated PDCD4. (a) Fluorescence images (×100) showed RAW264.7 cells were infected with SOCS1 overexpression adenovirus (Adv. SOCS1) and vector adenovirus (Adv. GFP). (b) qPCR detected the SOCS1 mRNA expression after cells were infected with Adv. SOCS1 and Adv. GFP for 48 h: ^*∗*^ *p* < 0.05 versus control, ^∧^ *p* < 0.05 versus Adv. GFP group. (c) Downregulation of the expression of STAT3 mRNA by STAT3 siRNA in macrophages RAW264.7 cells. The relative expression of STAT3 mRNA was detected by qPCR after 50pM STAT3 siRNA, including si-01, si-02, and si-03, and negative control (NC) was transfected into RAW264.7 cells for 48 h, respectively: ^*∗*^ *p* < 0.05 versus control, ^∧^ *p* < 0.05 versus NC group. (d) Western blot analyzed the protein level of phospho-STAT3 (p-STAT3) when RAW264.7 cells had been treated by 50pM STAT3 siRNA (si-03) and NC for 48 h. Representative bands show the protein expression of STAT3 (the upper bands), *β*-actin (the lower bands) in left panel, and the histograms in right panel showing quantification of STAT3 bands' normalization relative to *β*-actin expression in (d). ^*∗*^ *p* < 0.05 versus NC group. (e) Western blot analyzed the protein level of SOCS1, p-STAT3, and PDCD4 in RAW264.7 cells. They were transfected into the Adv. SOCS1 or STAT3 siRNA for 24 h and then treated by ox-LDL for 24 h. Representative bands show the protein expression of SOCS1 (the upper bands), p-STAT3 (the middle bands), PDCD4 (the third bands), and *β*-actin (the lower bands) in left panel, and the histograms in right panel showing quantification of STAT3 bands' normalization relative to *β*-actin expression. ^*∗*^ *p* < 0.05 versus control, ^∧^ *p* < 0.05 versus 20 *μ*g/mL ox-LDL group, and ^#^ *p* < 0.05 versus 20 *μ*g/mL ox-LDL plus Adv. SOCS1 group. The data are presented as the mean ± SE of three separate experiments.

To further examine whether miR-155 regulated PDCD4 via the SOCS1-STAT3 pathway, Raw264.7 cells were infected with SOCS1 overexpression adenovirus or transfected with STAT3 siRNA (Figures 6(a) to 6(d)). Expression of PDCD4 was increased by ox-LDL treatment and downregulated by SOCS1 overexpression adenovirus or STAT3 siRNA (Figure 6(e)). Taken together, this suggests that miR-155 regulated PDCD4 expression via the SOCS1-STAT3 pathway.

### 3.6. Downregulation of miR-155 Inhibited Inflammation Response against Atherosclerosis

**Figure 7:**
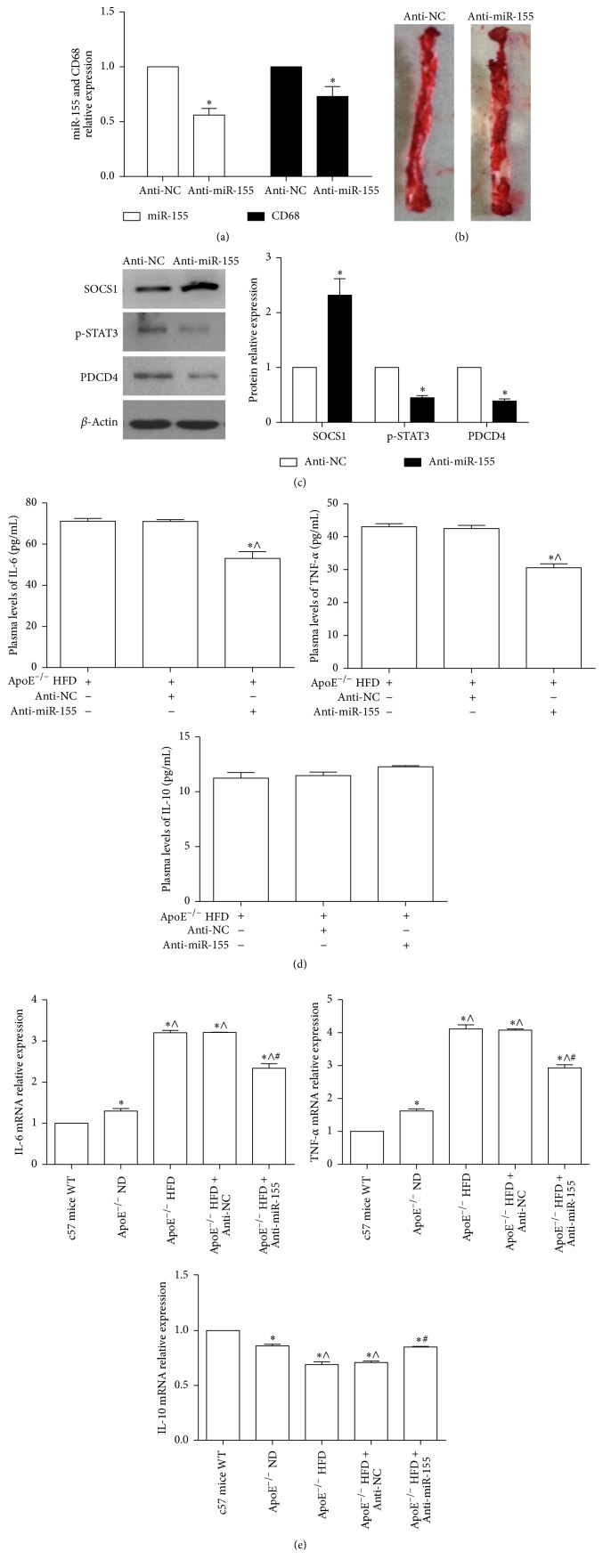
Inhibition of miR-155 by Anti-miR-155 decreased the atherosclerotic development in ApoE^−/−^ mice with HFD (ApoE^−/−^ HFD mice). (a) qPCR detected the miR-155 and CD68 mRNA expression in aortic tissue when ApoE^−/−^ HFD mice were injected with anti-miR-155 and anti-NC. ^*∗*^ *p* < 0.05 versus anti-NC. (b) Atherosclerotic plaques in the thoracoabdominal aorta of anti-miR-155 and anti-NC-injected ApoE^−/−^ HFD mice were assessed by Oil Red O staining, and the plaques areas were quantified by using Imagepro-plus 7.0: ^*∗*^ *p* < 0.05 versus anti-NC. (c) Western blot analyzed the protein level of SOCS1, p-STAT3, and PDCD4 in aortic tissue when ApoE^−/−^ HFD mice were injected with anti-miR-155 and anti-NC for 2 weeks. Representative bands show the protein expression of SOCS1 (the upper bands), p-STAT3 (the middle bands), PDCD4 (the third bands), and *β*-actin (the lower bands) in the left panel, and the histograms in the right panel show quantification of the bands' normalization relative to *β*-actin expression in (c). The data are presented as the mean ± SE of three separate experiments: ^*∗*^ *p* < 0.05 versus anti-NC. (d) The plasma level of IL-6, TNF-*α*, and IL-10 of anti-miR-155 and anti-NC-injected ApoE^−/−^ HFD was detected by the ELISA kit (*n* = 4): ^*∗*^ *p* < 0.05 versus ApoE^−/−^ HFD group, ^∧^ *p* < 0.05 versus anti-NC-injected ApoE^−/−^ HFD group. (e) qPCR analyzed the expression of IL-6, TNF-*α*, and IL-10 of c57 mice, ApoE^−/−^ ND, ApoE^−/−^ HFD, anti-miR-155, and anti-NC-injected ApoE^−/−^ HFD mice in aortic tissue (*n* = 4): ^*∗*^ *p* < 0.05, relative to c57 mice WT, ^∧^ *p* < 0.05, relative to ApoE^−/−^ ND, ^#^ *p* < 0.05, relative to ApoE^−/−^ HFD.

To address the role of miR-155 in the inflammation response of atherosclerosis formation in vitro, several male ApoE^−/−^ mice were fed an HFD and then injected with anti-NC or anti-miR-155 via the tail vein. These data showed that the expression of miR-155 and CD68, a marker of macrophages, was notably decreased in aortic tissue when ApoE^−/−^ HFD mice were injected with anti-miR-155 (Figure 7(a)). More importantly, the area of atherosclerotic plaques was obviously decreased in the anti-miR-155-injected ApoE^−/−^ HFD mice (Figure 7(b)). Western blot results showed that the SOCS1 protein increased in the aortic tissue of the anti-miR-155-injected ApoE^−/−^ HFD mice, whereas the PDCD4 and p-STAT3 protein expression obviously decreased (Figure 7(c)). Additionally, ELISA results showed that the plasma level of TNF-*α* and IL-6 increased significantly in anti-miR-155-injected ApoE^−/−^ HFD mice; however, the plasma IL-10 levels showed no difference between the anti-NC-injected and anti-miR-155-injected ApoE^−/−^ HFD mice (Figure 7(d)). qPCR analysis showed that the level of TNF-*α* and IL-6 mRNA increased significantly in ApoE^−/−^ HFD mice and in those that were suppressed in anti-miR-155-injected ApoE^−/−^ HFD mice (Figure 7(e)). On the contrary, the expression of IL-10 mRNA was reduced in ApoE^−/−^ HFD mice, and it could be partly reversed in anti-miR-155-injected ApoE^−/−^ HFD mice. These data suggested that miR-155 regulates the inflammation response in the atherosclerosis formation, which might be through SOCS1-STAT3-PDCD4 axis.

## 4. Discussion

In this study, we reported that inhibition of miR-155 could partially stunt the inflammation response in atherosclerotic development. We have proved that miR-155 regulates PDCD4 expression via directly mediated SOCS1 and its downstream STAT3 in vitro. Moreover, knocked down miR-155 considerably relieved the atherosclerotic plaques in the ApoE^−/−^ mouse model.

PDCD4 is involved in apoptosis, inflammation, and terminal differentiation [22, 39, 40]. The present study showed that PDCD4 increased with the elevation of IL-6 and TNF-*α*, and IL-10 was suppressed after macrophages were exposed to ox-LDL. On the contrary, knocked down PDCD4 by siRNA would inhibit the production of IL-6 and TNF-*α* and improve IL-10 expression. This result was consistent with Liang's report, which showed that downregulated PDCD4 suppressed the expression of proinflammatory factors and promoted the production the anti-inflammatory factor, IL-10 [24]. Other studies suggest that the PDCD4/nuclear factor-*κ*B/tumor necrosis factor *α* (PDCD4/NF-*κ*B/TNF-*α*) signaling pathway plays an important role in coronary microembolization- (CME-) induced inflammation, and inhibition of PDCD4 could improve CME-induced cardiac dysfunction [41]. Moreover, PDCD4 improved the inflammatory response via nuclear factor-*κ*B (NF-*κ*B), activating and inhibiting the production of IL-10 [24].

Recent studies have shown that several transcriptional factors, including AP-1, C-myb, and NF-kB, upregulated the expression of miR-155 in the immune system [42, 43]. In our study, we also found that PDCD4 was regulated by miR-155; therefore, it suggests that PDCD4 played an important role in miR-155 regulation of NF-kB and production of inflammation mediators. However, the manner used to regulate PDCD4 by miR-155 is unclear. SOCS1 played an important role in the inhibition of inflammation response via suppression of the JAK-STAT pathway [44]. Several studies found that SOCS1 was a function target of miR-155 signaling from these *γ*-chain cytokine receptors [45]. Previous studies have demonstrated the crucial role of miR-155 in regulating CD8 T-cell responses by targeting SOCS1 to ensure *γ*-chain cytokine signaling [46]. miR-155 deficiency causes defective expansion of effector CD8 T-cells and the generation of memory CD8 T-cells due to increased SOCS1 expression [46]. STAT3 signaling, particularly the constitutive activation of STAT3, is important in development and carcinogenesis, since it critically regulates the transcription of multiple key genes involved in cell proliferation, differentiation, apoptosis, angiogenesis, immune response, and metastasis [47, 48]. Activation of STAT3 has been shown to upregulate the expression levels of PDCD4, PTEN, and RECK [49]. Our study showed that SOCS1 overexpression and downregulation of STAT3 would mediate the PDCD4 expression. Taken together, these findings suggest that miR-155 mediated PDCD4 via the SOCS1-STAT3 pathway which regulates the inflammation response in RAW 264.7 cells.

This study shows that circulating miR-155 was increased significantly in the plasma of HFD-fed ApoE^−/−^ mice, in which early atherosclerotic plaques had already formed in the aortic roots. Further results indicate that circulating miR-155 in the plasma could be released from activated macrophages. In vivo silencing of miR-155 in mice by injection of antagomiR-155 attenuated levels of proinflammation factors in the plasma and aortic tissue and in the formation of atherosclerotic plaques in atherosclerotic mice. Moreover, antagomiR-155 upregulated the SOCS1 protein and downregulated the expression of PDCD4 and p-STAT3. These data revealed that miR-155 might regulate the inflammation response and atherogenesis through the SOCS1-STAT3-PDCD4 axis.

In conclusion, miR-155 mediated the SOCS1-STAT3-PDCD4 axis to regulate the production and release of inflammation mediators and played a critical role in the formation of atherogenesis. It would serve as a novel target for atherosclerotic disease treatment.

## Supplementary Material

(1) The siRNA sequences of mmu-STAT3 and mmu-PDCD4 were designed and synthesized by Guangzhou sharp RIBOBIO biological technology co ., LTD. They provided siRNA sequences for knocking down the mRNA expression of STAT3 and PDCD4 in three target site, respectively (Suppl Table 1). To screen out the optimal siRNA sequence for further study, these sequences were transfected into RAW 264.7 cells by lipofiter regents for 48h, and the expression of STAT3 and PDCD4 was detected by RT-qPCR.
(2) The mRNA expression of mmu-miR-155, SCOS1, STAT3 and PDCD4 was detected by RT-qPCR. U6 was severed as the control of miRNA. The primer detail as shown at Supplemental Table 3.

## Abbreviations

AS: Atherosclerosis
PDCD4: Programmed cell death protein 4
STAT3: Signal transducer and activator of transcription 3
SOCS1: Suppressor of cytokine signaling 1
IL-10: Interleukin-10
IL-6: Interleukin-6
TNF-*α*: Tumor necrosis factor-*α*
ND: Normal diets
HFD: High fat diets.
