# Supplementary material for: miR-155 Regulated Inflammation Response by the SOCS1-STAT3-PDCD4 Axis in Atherogenesis
Source: Mediators Inflamm. 2016 Oct 24;2016:8060182. doi: 10.1155/2016/8060182 (PMC5098093; doi:10.1155/2016/8060182)
Supplement: Supplementary file 1 — (1) The siRNA sequences of mmu-STAT3 and mmu-PDCD4 were designed and synthesized by Guangzhou sharp RIBOBIO biological technology co ., LTD. They provided siRNA sequences for knocking down the mRNA expression of STAT3 and PDCD4 in three target site, respectively (Suppl Table 1). To screen out the optimal siRNA sequence for further study, these sequences were transfected into RAW 264.7 cells by lipofiter regents for 48h, and the expression of STAT3 and PDCD4 was detected by RT-qPCR. (2) The mRNA expression of mmu-miR-155, SCOS1, STAT3 and PDCD4 was detected by RT-qPCR. U6 was severed as the control of miRNA. The primer detail as shown at Supplemental Table 3. [file 8060182.f1.docx]

**Supplemental Table 1** Target site of mmu-STAT3 siRNA

| No. | Product number | Target site suquence |
| --- | --- | --- |
| 01 | siG111226111821 | CCACGTTGGTGTTTCATAA |
| 02 | siG111226111834 | GCAGGATCTAGAACAGAAA |
| 03 | siG111226111845 | GCATCAATCCTGTGGTATA |

**Supplemental Table 2** Target site of mmu-PDCD4 siRNA

| No. | Product number | Target site suquence |
| --- | --- | --- |
| 01 | siG150915034903 | CCAGGAGAACTGTGTTTAT |
| 02 | siG150915034910 | GCTCCTGAGTATGTCCAAA |
| 03 | siG150915034919 | CCCACACTCATACTCTGTT |

**Supplemental Table 3** Real-time PCR primers, products size and conditions

| No. | Gene | Primer Sequence (5’→3’) | Product Length | Annealing Temperature |
| --- | --- | --- | --- | --- |
| 1 | mmu-miR-155-RT | 5'CTCAACTGGTGTCGTGGAGTCGGCAATTCAGTTGAGACCCCTAT3' |  |  |
| 2 | mmu-miR-155 | F:5'ACACTCCAGCTGGGTTAATGCTAATCGTG3' R:5'CTCAACTGGTGTCGTGGAGT3' |  | 60 ℃ |
| 3 | mmu-U6-RT | 5'CAAAATATGGAACGCTTC**3'** |  |  |
| 4 | mmu-U6 | F:5'GTGCTCGCTTCGGCAGCA3'  R:5'CAAAATATGGAACGCTTC3' |  | 60 ℃ |
| 5 | SOCS1 | F:5’ GATTCTGCGTGCCGCTCT3' R:5' TGCGTGCTACCATCCTACTC 3' | 153 bp | 60 ℃ |
| 6 | STAT3 | F:5’TATCTTGGCCCTTTGGAATG3' R:5'CTGAAGCGCAGTAGGAAGGT3' | 108bp | 60 ℃ |
| 7 | PDCD4 | F:5' AACAAAACAAAAACAAACAGAGCG3' R:5TTAATTTCGCTTGCACTGA3' | 180 bp | 60 ℃ |
| 8 | β-actin | F:5'CACGGCATCGTCACCAACT3'  R:5'GTCCTACGGAAAACGGCAGA3' | 251 bp | 60 ℃ |
